# Book Reviews

**Published:** 1898-01

**Authors:** 


### BOOK REVIEWS.

A Practical Treatise on Diseases of the Skin. John V. Shoemaker, M.D., LL.D., Professor of Skin and Venereal Diseases in the Medico-Chirurgical College and Hospital of Philadelphia. Physician to the Philadelphia Hospital for Diseases of the Skin, etc., etc. Third edition. Revised and enlarged, with chromogravure plates and other illustrations. New York, D. Appleton & Co., 1897.

The bibliography of the author is very full and the text shows extensive knowledge of cutaneous literature. The indexes are very complete. The illustrations are not over-numerous, but are of good quality.

Some of the newer diseases are described, others are omitted. Many disease conditions which are usually left out of such works are found in this, as chancroid, for example.

"Xeroderma pigmentosum," a case of which we have published, does not seem properly to belong under the head of atrophies, as commonly placed. Atrophy is a symptom, but pigmentations, telangiectases and new growths are equally typical, if not necessary, to the diagnosis of the disease. Its symptom-complex renders it difficult of classification.

Erythema, or redness, is given as one of the primary lesions in skin diseases. This is usually covered by the "macule," or an extension of the term.

The chapter on general diagnosis is very instructive and practical. Under "odor" he might have described that so often characterizing late ulcerating syphilis and that of destructive carcinoma.

In speaking of arsenic, he expresses a preference for arsenious acid, or sodium arseniate, as more reliable than the ready-made solutions. He lauds chlorate of potassium internally, as useful in suppurative conditions and cases giving evidence of "suboxidation." Baths, soaps, fats, ointments, pastes, and oleates are very fully treated, and the whole matter of treatment is handled in the manner which we should expect of so good a practitioner. Many applications and drugs are described which are usually omitted in similar works. In fact, the author shows a greater belief than the average physician in the virtues of drugs.

Comedo is handled as an anomaly of secretion.

His hyperemias are limited to erythema simplex and erythema intertrigo.

Lupus erythematosus, and several other diseases which we are accustomed to look upon as not of the nature of new growths, typical or atypical of the structures in which they lie, are classed under tumors.

Formulæ of use in the various diseases are given in the text, and a full formulary appears in the end.

It is impossible in our space to go through and comment upon the whole book, so it must suffice to say that it is complete, thorough and practical enough to meet the needs of almost any practitioner.

M. B. H.

---

THE LIVING SUBSTANCE AS SUCH, AND AS ORGANISM. By Gwendolen Foulke Andrews (Mrs. Ethan Allen Andrews). Supplement to *Journal of Morphology*, Vol. XII., No. 2. Boston. Ginn & Co. The Atheneum Press. 1897. Price $1.50.

This book is certainly not lacking in ambition. Mrs. Andrews' researches have brought her to the conclusion that heretofore biologists have been working from an altogether erroneous standpoint, and these pages, in no very humble mind, she has written to put them right. "To concern one's self much with the living substance as such, with the habit of its substance rather than its form; to see, to understand, how the substance expresses itself in relation to its environment, both general and specific; to follow it in its complex action and reaction; to learn how it uses, rejects, transcends, or passively protects itself against external conditions; and how it bends itself before these in order to conquer by a magnificent 'jiujutsu,' this seems to me the true biological standpoint of the living substance as such: it is the working standpoint of nature." The authoress hopes by her work to turn "the biologist from the artificial, the dogmatic, the systematic mode of study," into the pursuit of the substance life, substance structure, and, above all, substance habit. He will then allow his microtome and paraffin bath to lie and rest. She holds that the structures and functions, visible and invisible, of the various forms of animal life, do not express their individuality, and that they are but incidental and minute, local, vesicular organization and function of a protoplastic ground substance; "that the zoological or botanical unit is in part a psychological formulation of mere separateness, while actually a vesicular accident or functional incident of the substance organism's protoplastic life." We doubt, from what we have been able to read of this book, whether prospectors after truth will look upon it as "pay dirt"; the gold does not lie on the surface, and requires a deal of work for its extraction. The volume is nicely printed and bound. A. W. S.

---

DISEASES OF THE EYE. By Edward Nettleship, F.R.C.S., Ophthalmic Surgeon at St. Thomas's Hospital, London; Surgeon to the Royal London (Moorfields) Ophthalmic Hospital. Revised and edited by W. T. Holmes Spicer, M.A., M.B., F.R.C.S., Ophthalmic Surgeon to the Metropolitan Hospital and to the Victoria Hospital for Children. With a supplement on Color Blindness by William Thomson, M.D., Emeritus Professor of Ophthalmology in the Jefferson Medical College of Philadelphia. Handsome 12mo of 521 pages, with 2 colored plates and 161 engravings. Cloth, $2.25. Lea Brothers & Co., Publishers, Philadelphia and New York.

This book has long been a favorite with students, as well as with practitioners who desire a compact treatise on diseases of the eye. The present is the sixth edition, and in its preparation the former works of Nettleship have been carefully revised by Mr. Holmes Spicer. A useful supplement on "The Practical Examination of Railway Employés as to Color Blindness, Acuteness of Vision and Hearing" has been added by Professor William Thomson, of Philadelphia. An appendix containing the more common formulæ used in eye diseases closes the volume. In spite of frequent revisions some errors remain in proof-reading and cross-references. On page 137 "F. 12 and 13" should be "25 and 26," "F. 14" should be "27."

For its size there is within our knowledge no better book on diseases of the eye than Nettleship's, and we take pleasure in recommending it. It is believed that readers of this edition will find it an epitome of the best ophthalmology of the present time.

---

LECTURES ON THE MALARIAL FEVERS. By William S. Thayer, M.D., Associate Professor of Medicine in Johns Hopkins University, Baltimore. D. Appleton & Co., New York. [Price, $3.]

Than malarial fevers no class of diseases is of greater interest to the general practitioner of medicine. In the South physicians in country or city are almost daily summoned to see some form of malarial intoxication. If he will read this volume of lectures by Dr. Thayer, no point, either biological, prophylactic, clinical, or therapeutic, will escape his notice. The biological history of the plasmodium malariæ, as well as the biology, is a happy epitome of the international investigations. The author, while exhaustively portraying the efforts of others, modestly places in the background his own valuable researches.

The objective teaching per fever chart is very instructive.

The plates, picturing the plasmodium in different stages of development and decadence, are beautiful, artistic, and very instructive.

Then the differential diagnosis of the clinical forms of the malarial fevers is, beyond doubt, a masterful effort.

The careful study of these lectures has proven a source of much knowledge, and we feel assured they offer the same to any reader.

The book is of convenient size and well printed. H. H.

---

A TEXT-BOOK FOR TRAINING-SCHOOLS FOR NURSES. By P. M. Wise, M.D., President of New York State Commission of Lunacy, Albany. Introduction by Dr. Edward Cowles, Boston. Two volumes. Each $1.25. G. P. Putnam Sons, 27 W. 23d St., New York.

For a long time there has been a need for a systematized course of instruction for nurses during their course in the training schools. There are many of these books extant which have been simplified and condensed so that their usefulness has been largely marred. In the text-book before us we find an unusually fortunate arrangement; this is comprehensive enough to embrace most of the essentials of nursing and is so arranged that the student advances by regular lessons from the simplest consideration of the work to the most complex. This work is in two volumes, the first being largely devoted to a consideration of the tissues of the body, the food and its digestion, some of the most common micro-organisms, the care and attention to the room and bed, etc. This volume endeavors to prepare the student for a proper appreciation of the second which enters fully into the details of nursing and separates the chapters upon surgical and medical cases, and further describes the special care necessary for nursing children, the various febrile diseases, cases of insanity, etc.

Throughout the entire book the coöperation of the nurse with the physician is insisted upon, and the reliance which the former must place upon the latter is well brought out.

This is a most excellent book of its kind, and will find a most hearty welcome in the schools and hospitals devoted to the training of nurses. E. C. D.

---

ABOUT CHILDREN: Six lectures given to the nurses in the Training School of the Cleveland General Hospital. By Samuel W. Kelley, M.D., Professor of Diseases of Children in the Cleveland College of Physicians and Surgeons. In Buckram, 180 pages, postpaid, $1.25. The Medical Gazette Publishing Company, Cleveland, Ohio.

This is the title of a series of lectures delivered to the Training School for Nurses by Dr. Kelley upon a branch of nursing which differs materially from the nursing of the adult, and is often overlooked by the authors of books upon nursing. In this brief course of lectures the author endeavors in a concise way, first, to show wherein the child differs from the adult in the structure of its tissues and in the care essential to successful nursing. There are many apparently insignificant details connected with the nursing of children during their illness, the regulation of the dietary, the handling and care of children, which needs special mention. This is all done in a very successful manner by the author, making the little volume interesting as well as useful. E. C. D.

---

CONSTIPATION IN ADULTS AND CHILDREN. With Special Reference to Habitual Constipation, and its Most Successful Treatment by the Mechanical Methods. By H. Illoway, M.D. Pages 495. The MacMillan Company, Publishers, New York. Price, Cloth, $4.00.

This work treats of a very important and too often neglected subject—the source of an enormous amount of discomfort in the aggregate, and of many bodily ills.

The several chapters are devoted to consideration of the anatomy of the intestines, to flatus, peristalsis, fæces, to the etiology of constipation, its classification, symptomatology, consequences and treatment.

Special stress is laid upon the value of mechanical means of treatment, and the various methods of applying abdominal massage are fully described and freely illustrated. Hygiene, diet and exercise receive deserved attention as important elements in the rational treatment of this affection. Hydrotheraphy, the use of electricity and the technique of oil and of other rectal injections are clearly explained.

The suggestions presented are, for the most part, practical, and if the author magnifies the importance of special mechanical treatment and underestimates the worth of drugs, a slight allowance in both directions will suffice to correct a tendency which may impair but does not destroy the value of the book.

Where there is much to commend, exacting criticism of minor points and of palpable but comparatively trivial defects, here and there, appears to be unnecessary and unprofitable.

The style of the writer is not classical, but it is sufficiently direct and explicit for easy comprehension, and the text is abundantly illustrated.

The typography and general make-up of the book are excellent.

J. B. B.

---

THE LOFOTEN ISLANDS AND THEIR PRINCIPAL PRODUCT. Detroit. Parke, Davis & Co. 1897.

This is a beautifully printed and illustrated booklet, which gives a description of these islands, Norway, to which they belong, and the cod-fishing industry. A good part of the book is devoted to Norway, its climate and people, but especial attention is given the fisheries, as found in the Lofoten Islands, the fishermen, boats, etc. The details of the preparation of the fish and their livers are given. The matter and the illustrations are very interesting. The book can be had gratis from the publishers.

---

COLLIER'S WEEKLY, DECEMBER 16, 1897.

AN able editorial on the spirit and scope of the President's Message, with a scholarly discussion of Speaker Reed's recent dictum, "*Empire can wait,*" add weight and interest to the current number of COLLIER'S WEEKLY. The crisp paragraphs of Saltus, the indefatigable newsgathering industry of Habberton, the philosophic comments of Hawthorne and the eminently readable London letter of Edgar Fawcett, are attractions already sufficiently known to readers of this brilliant journal. The Editor promises in addition some startling innovations with the new year. Among them is a project long cherished of giving weekly, in connection with paper proper, a literary magazine. The inauguration of various new and important departments is also promised, and last, but by no means least, Henry James's serial story, "The Turn of the Screw."

